# A Prospective Self-Report Survey-Based Cohort Study on Factors That Have an Influence on Tinnitus

**DOI:** 10.3390/audiolres14050074

**Published:** 2024-10-10

**Authors:** Jana V. P. Devos, Marcus L. F. Janssen, A. Miranda L. Janssen, Catharine A. Hellingman, Jasper V. Smit

**Affiliations:** 1 Mental Health and Neuroscience Research Institute, Maastricht University, 6229ER Maastricht, The Netherlands; jana.devos@maastrichtuniversity.nl (J.V.P.D.);; 2 Department of Ear, Nose, and Throat, Maastricht University Medical Center+, 6229HX Maastricht, The Netherlands; 3 Department of Clinical Neurophysiology, Maastricht University Medical Center+, 6229HX Maastricht, The Netherlands; 4 Department of Methodology and Statistics, CAPHRI Public Health Research Institute, Maastricht University, 6229ER Maastricht, The Netherlands; 5 Department of Ear, Nose, and Throat/Head and Neck Surgery, Zuyderland Medical Center, 6419PC Heerlen, The Netherlands

**Keywords:** tinnitus, cohort, demographics, comorbidities, personalized care

## Abstract

**Background**: Limited information is available on factors that affect the burden tinnitus. The aim of this study is to investigate the association between tinnitus burden and demographic, patient-specific and tinnitus characteristics. Secondly, it was examined which variables could predict a change in tinnitus burden after 12 months. **Method**: In a prospective Dutch cohort of 383 tinnitus patients seeking medical help, tinnitus complaints, demographics, tinnitus characteristics, psychological wellbeing and quality of life were assessed using an online self-report survey at three timepoints (start, 6 months, 12 months). The main outcome variables for tinnitus burden are the Tinnitus Questionnaire (TQ) and Visual Analog Scale (VAS) for tinnitus burden and loudness. **Results**: Several variables (time, sex, education level, life events, anxiety and depression, sleep issues, tinnitus loudness, hearing impairment and treatment) were significantly associated with tinnitus burden. Additionally, tinnitus burden after 12 months was associated with anxiety, following treatment, sleep issues, negative life events and hearing impairment (increase) and anxiety, total of life events and environmental quality of life (decrease) predicted the tinnitus burden after 12 months. **Conclusions**: Several factors, such as education level, life events, psychological factors and sleep quality, are related to tinnitus burden and can predict tinnitus burden over time.

## 1. Introduction

Tinnitus is the conscious awareness of a tonal or composite noise for which there is no identifiable corresponding external acoustic source [1]. Up to 15% of the general population experiences tinnitus [2]. It has been shown that countries with a higher gross domestic product have a lower tinnitus prevalence [3]. This study was conducted in the Netherlands; therefore, it is possible the prevalence is relatively low. The prevalence of tinnitus in the Netherlands is not known; however, in neighboring countries the prevalence is, respectively, 14.1% in France and 11.9% in Germany. The prevalence in the Dutch population is expected to be in this range. In 1% of the population, tinnitus has a large impact on several aspects of daily life [2], such as interference at work, issues in social interaction, emotional distress, sleep deprivation and decreased overall health [4,5,6]. Additionally, patients suffering from tinnitus are more prone to depression, anxiety and insomnia [5,7,8]). 

The neurophysiological model of tinnitus postulates that the auditory pathway is not the only system involved in the mechanism of tinnitus; there seems to be a major role for the limbic and autonomic nervous system [9]. The limbic system is primarily involved in emotional regulation, which has a considerable role in tinnitus burden. Evidence of the autonomic role in tinnitus is less established, yet tinnitus distress seems related to sympatic activation [10]. 

Tinnitus can be divided into objective and subjective tinnitus. In objective tinnitus, an underlying cause such as vascular stenosis or a myoclonus can be found. In the majority of tinnitus patients, no pathology other than some form of hearing loss could be found, which is then defined as subjective tinnitus [11]. In the case of objective hearing loss, audiological rehabilitation is recommended. A definitive treatment for objective tinnitus is rare and in most cases the same steps are taken after diagnosis, namely reassurance and education on the underlying mechanisms of tinnitus. Especially in the population of people who actively seek help for their tinnitus burden these mechanisms are of interest. Information on tinnitus predictors is crucial to develop individualized counseling strategies. To date, only limited information is available on factors that positively or negatively impact tinnitus burden as previous studies are of variable quality with poor tinnitus definitions and evaluations or questionable sampling of the study population as the main factors contributing to a high risk of bias [12]. Nonetheless, several risk factors have been identified, including cardiovascular, psychological, neurological, musculoskeletal and dietary factors. In a more recent study, higher levels of somatization and a history of smoking were identified as risk factors for developing new-onset tinnitus over a 5-year period. Additionally, anxiety and poor speech recognition in noisy environments were linked to greater annoyance from new-onset tinnitus [13]. 

The aim of this study was twofold. Firstly, the relationship between demographic and patient-specific factors and tinnitus burden was studied. Secondly, the impact of demographic and patient-specific factors on tinnitus burden over one year was measured. To this end, we have set up a prospective self-report survey study in a help-seeking tinnitus population to explore characteristics that are associated with tinnitus burden. 

## 2. Methods

### 2.1. Study Design

This longitudinal self-report survey study was designed to assess the demographics, tinnitus characteristics and psychosocial influence of a help-seeking tinnitus population. The medical ethical committee (METC/institutional review board) of Maastricht University Medical Center provided approval to conduct the study (2019-1413).

### 2.2. Recruitment and Informed Consent

Patients suffering from tinnitus were recruited by (e-)mail after a visit at the outpatient clinic of the ear, nose and throat/audiology department of a secondary care hospital (Zuyderland Medical Center, Heerlen, The Netherlands) and a tertiary care hospital (Maastricht University Medical Center+, Maastricht, The Netherlands). Only one email was sent to participants and participants could only sign up independently and anonymously. Recruitment started 1 July 2020 and was closed 1 July 2021. 

### 2.3. Inclusion and Exclusion Criteria

Adults suffering from tinnitus consistently (at least more than once a week, both acute (<6 months) and chronic (>6 months)) who sought help at the outpatient clinic of the ear, nose and throat department were eligible to take part in the study. It is important to note that, in The Netherlands, patients can only be referred to an otolaryngologist and/or audiological center by their general practitioner (GP), they cannot make an appointment on their own initiative. This means referrals are already filtered by a medical doctor, namely the GP. Persons that dropped out of the study before the final assessment were excluded from the analysis. 

### 2.4. Data Acquisition

There was no permission sought to access any patient records and all data are self-reported. Questionnaires were provided using the online questionnaire software Qualtrics [14]. The questionnaires provided to patients remained available for two weeks. The median time to fill out the questionnaire was 40 min. The self-report survey was designed in such a way that no missing data within a participant per follow-up moment was possible. The questionnaire was repeated after 6 and 12 months. 

### 2.5. Questionnaires

The self-report survey was composed of several sections. First demographics and general health were addressed. Next, tinnitus complaints and perceived hearing abilities were evaluated. The Tinnitus Questionnaire (TQ) [15,16] was used to assess tinnitus severity. A clinically relevant change is defined as a change of ≥11 points of the TQ score. TQ scores can be divided into 4 grades of severity: grade 1 (little burden, 0–30 points), grade 2 (mild burden, 31–46 points), grade 3 (severe burden, 47–59 points) and grade 4 (very severe burden, 60–84 points) [17]. Visual Analog Scales (VAS) [18,19] were used to assess loudness and the burden of the tinnitus. Anxiety and depression were evaluated using the Hospital Anxiety and Depression Scale (HADS) [20,21]. Quality of Life (QoL) was assessed using the WHO-QOL BREF [22]. Lastly, help-seeking behavior and life events and their impact were evaluated. See Appendix A for a detailed description of the questionnaires used.

### 2.6. Data Processing and Statistics 

The data were analyzed using IBM SPSS Statistics [23] version 25. The results are presented using the mean (*M*) and standard deviation (*SD*), unless stated otherwise. 

To assess the correlation between continuous variables, Pearson’s correlation was used. In case of multiple comparisons, the *p*-value was adjusted using a Bonferroni correction. To compare means an independent T-test was used. To compare the means at baseline and after 12 months a paired samples T-test was used. 

Since every participant was measured at three time points, the assumption of independence was violated. Therefore, the relationship between demographic and patient-specific factors and tinnitus burden (estimated by total TQ score) was investigated using marginal linear regression analyses with an unstructured covariance matrix of the residuals. To assess the role of several other factors on tinnitus burden and to adjust for possible confounding, the regression model included in addition to time (0, 6, and 12 months) the following variables: sex (male, female), number of life events, age at baseline, education level at baseline (low/high), treatment (yes/no) and HADS anxiety scores, HADS depression scores, VAS loudness, and the presence of sleep problems at all timepoints. The normality assumption of the regression analysis has been checked by a histogram and a normal P-P plot of the residuals.

To assess the impact of demographic and patient-specific factors on tinnitus burden over one year, first all demographic variables (sex, education, age) and patient-specific variables (tinnitus duration, VAS loudness at baseline, HADS anxiety, HADS depression, sleeping issues reported between T0 and T2, amount of life events, QoL and its sub scores) were tested using simple regression analysis. Based on the univariable linear regression analysis variables with a *p* > 0.2 were excluded. The variable sex and burden at baseline were added based on relevance even though *p* < 0.2. With this set of variables, a backward linear regression analysis was performed eliminating each variable that was not significant (*p* < 0.05). 

Treatments that were considered effective are audiological care, cognitive behavioral therapy and medication for psychiatric comorbidities [24,25,26,27,28,29].

Education level was dichotomized from the original six levels into education not extending past high school (low, Verhage ≤ 5) and education extending past high school (high, Verhage > 5) [30]. The Verhage system is a way of dividing education level based on length and type of schooling generally used in the Netherlands, Verhage > 5 includes higher professional education and university education. For all statistical analysis α was set at 0.05.

## 3. Results

### 3.1. Study Participants

A total of 452 patients completed the first online questionnaire. Out of all 452 participants 93% (*n* = 424) filled out the questionnaire after 6 months (T1) and 90% (*n* = 383) filled out the 12 months follow-up (T2). 

The cohort consisted of 60% males and 40% females. The mean age at baseline was 57 years (*SD* = 13), ranging from 18 to 88 years. The median duration of tinnitus was 6 years ranging between 0.2 and 61 years at T0. Demographics at baseline are summarized in Table 1. 

About half the participants had a low education (51%) and approximately half of the participants were working, either full-time (35%) or part-time (13%). In total, 26% of the participants were retired. The percentage of participants that were not working due to chronic illness was 6%; in one-third of the participants not working due to a chronic illness (2%), this was due to their tinnitus. This last group (participants not working due to tinnitus) had a relatively high tinnitus burden (*M* = 60, *SD* = 9) compared to the complete cohort (*M* = 34, *SD* = 18, *p* < 0.001) and perceived tinnitus to be relatively loud (VAS loudness *M* = 85, *SD* = 8), as compared to the full population (*M* = 65, *SD* = 21, *p* < 0.001). Participants reported on various other medical conditions and procedures as part of their medical history (Appendix B). Most commonly, participants reported sleep issues (*n* = 191), cardiovascular conditions (*n* = 132) and psychological issues (*n* = 105). 

### 3.2. Tinnitus Scores

The mean tinnitus burden scores in this population were almost similar at all timepoints (TQ: baseline *M* = 34; 12 months *M* = 32). Although this difference was statistically significant (*p* = 0.017) it is not clinically relevant (see Table 2). 

As expected, tinnitus loudness as measured by the VAS had a significant association with TQ (*p* < 0.001). There is also a strong association (*r* = 0.8, *p* < 0.001) between the TQ score and the VAS burden score at baseline. This association was also present at 6 months (*r* = 0.8, *p* < 0.001) and 12 months (*r* = 0.7, *p* < 0.001).

### 3.3. Tinnitus Characteristics and Hearing

The median duration of tinnitus at baseline in this population was 6 years and 49% tinnitus had a sudden onset. There was no significant difference in tinnitus burden for participants who had a sudden or a gradual onset of tinnitus (*p* = 0.791). There were two reported major causes of tinnitus assumed by the participants: stress (reported 127 times) and noise exposure (reported 120 times). The majority of the participants reported hearing a single sound (57%). Almost all participants (95%) heard their tinnitus constantly. Most participants described the tinnitus sound as a high pitch sound (68%). The sound was most often described as a tone (59%) or as noise (28.5%), often (55%) with a variation in the sound. For 56% of participants the tinnitus was bilateral. Tinnitus was more common in predominantly the left ear (33%) than the right ear (27%). 

A large portion of the participants indicated that they perceive their hearing ability as being impaired (62%) which commonly was reported to be confirmed by a medical specialist with audiometry (89%). Participants that reported impaired hearing had a significantly (*p* < 0.001) higher TQ. Hearing devices were worn in 31% of all participants. 

For a complete overview of tinnitus characteristics at baseline, see Table 2.

The relationship of demographics and patient-specific factors with tinnitus burden (Table 3).

### 3.4. Impact of Demographic and Patient-Specific Factors on Tinnitus Burden 

A significant difference (*B* = −0.910) over time (per six months) in TQ score (*p* = 0.019) was observed. It is, however, important to note that a clinically relevant change is defined as a reduction of ≥11 points of the TQ score. On a group level, therefore, no clinically relevant change was observed. 

When having a closer look at the changes from one grade to another over time, a huge heterogeneity was observed (see Appendix C).

(a) Sex and age

In this cohort of tinnitus patients, men experienced a significantly (*p* < 0.001) higher tinnitus burden than women. No significant effect of age was observed on TQ score (*p* = 0.708).

(b) Education level

Participants with a higher education level had a significantly lower TQ (*p* < 0.001) compared to participants with a lower education.

(c) Life events

Both the total amount of positive (*p* = 0.029) and negative (*p* = 0.001) life events are significantly related to TQ score. Examples of the most reported life events are changes in sleep, changes in leisure and changes in social contacts.

(d) Anxiety and depression

Both anxiety and depression as measured by the HADS have a significant effect on the TQ score (relatively *p* < 0.001; *p* < 0.001).

(e) Treatment

In total, 28.8% of the participants reported having followed a treatment plan for their tinnitus in the observed period. This is a self-report of having followed a treatment plan with possible options being medical, audiological or psychological care, physiotherapy and other treatments the participants could add. The psychological, audiological and psychiatric treatments were labeled as being proven effective treatments; others were labeled as not proven effective. Both types of treatment were significantly related to TQ score (respectively *p* < 0.001; *p* = 0.010). Other treatments that were reported are, for example, physiotherapy, acupuncture and neuromodulation techniques.

(f) Psychological comorbidities and sleep

Psychological comorbidities were common (26%); participants reported anxiety (11%) and depressive symptoms (14%) most frequently.

Participants with reported psychological comorbidities (*M* = 41, *SD* = 18) scored significantly higher on the TQ (*p* < 0.001) than participants without (*M* = 30; *SD* = 17). 

Half of the participants (51%) reported having issues sleeping (either falling asleep, sleeping through the night or both). Participants with sleep issues (*M* = 40, *SD* = 18) scored higher on the TQ (*p* < 0.001) than participants without (*M* = 26, *SD* = 14). 

### 3.5. Predictors of Tinnitus Burden over Time

The original regression model included at baseline TQ, age, sex, education level, tinnitus duration in years, tinnitus loudness at baseline, HADS anxiety at baseline, HADS depression at baseline, treatment between T0 and T2 (yes/no), effectively proven treatment between T0 and T2 (yes/no), other treatment between T0 and T2 (yes/no), sleep issues reported between T0 and T2 (yes/no), total amount of life events reported between T0 and T2, amount of positive life events reported between T0 and T2, amount of negative life events reported between T0 and T2, QoL and its sub scores and hearing status (normal/impaired) as independent variables. TQ score at T2 was the dependent variable. 

The following variables were included in the final model after backward elimination of non-significant predictors: TQ at baseline, tinnitus duration in years, tinnitus loudness at baseline, HADS anxiety at baseline, effectively proven treatment between T0 and T2 (yes/no), other treatment between T0 and T2 (yes/no), sleep issues reported between T0 and T2 (yes/no), total amount of life events reported between T0 and T2, amount of negative life events reported between T0 and T2, QoL environmental subscore and hearing status (normal/impaired) as independent variables (see Table 4).

Evidently, the TQ at baseline is of significant importance (*p* < 0.001) in the prediction of the TQ after 12 months. The duration of the tinnitus (*p* < 0.001) and the perceived loudness of the tinnitus at baseline (*p* = 0.025) also predicted the burden outcome after 12 months. HADS anxiety at baseline was significant (*p* < 0.001) with, interestingly, higher anxiety scores predicting a lower tinnitus burden after 12 months. Surprisingly, having followed a treatment that was proven effective (*p* < 0.001) or another treatment (*p* < 0.001) between T0 and T2 predicted a higher TQ score after one year. Reporting sleeping issues was associated with a significant (*p* < 0.001) higher tinnitus burden after 12 months. The amount of negative life events reported to have occurred between baseline and 12 months were related to a significant (*p* < 0.001) higher tinnitus burden after 12 months. A better environmental sub score of the QoL measure at baseline was also significantly (*p* < 0.001) linked to a lower tinnitus burden after 12 months. Lastly, the presence of hearing impairment at baseline is related to significantly (*p* = 0.015) more tinnitus burden after 12 months. 

## 4. Discussion

In this prospective cohort study, several factors, such as hearing loss, education level, life events, psychological comorbidities and insomnia, are associated with tinnitus burden. As expected, TQ at baseline is linked to the TQ at 12 months [31]. In addition, the presence of anxiety (but not depressive symptoms) at baseline had a lowering impact on tinnitus burden after 12 months. Moreover, positive life events also alleviated tinnitus burden. Interestingly also environmental QoL is associated with a lower tinnitus burden after 12 months. 

When interpreting the results of this cohort, it is important to realize that all participants in this study visited the outpatient clinic. It is known that participants who seek help for their tinnitus have a high tinnitus burden with an impaired QoL [32]. This in turn can be linked to the stepped care protocol applied in the Netherlands where higher burden entails more extensive treatment. These results, for these reasons, simply cannot be extrapolated to the general tinnitus population.

### 4.1. The Relation of Demographic and Patient-Specific Factors and Tinnitus Burden

An interesting finding was that tinnitus burden was related to education level. In this cohort, participants with a higher education level reported lower burden scores. These results confirm findings from others [33,34]. It has also been shown that education level (and older age) are general risk factors for psychological distress [35]. Moreover, education level was identified to significantly contribute to a reduction in tinnitus burden after an online treatment program since having good literacy skills was essential when understanding the intervention materials [31]. These findings have impact on clinical care, as within international and Dutch tinnitus guidelines, one of the first steps in treatment is psycho-education. An issue could be that current psycho-education sessions are not well enough adapted to the individuals’ education level and consequently do not have the expected effect. This is an important note since it has been shown that adequate psycho-education can have a positive impact on tinnitus burden [36]. Additionally, the current standard care consists of audiological care and cognitive behavioral therapy (CBT) in which education level also seems important. Unfortunately, it was not possible to differentiate from the current data which participants only received psycho-education and which participants received CBT. 

Further, males reported a higher tinnitus burden measured by the TQ than females. In the literature, there is no consensus on differences in burden based on sex [37,38,39,40]. Tinnitus is known to be more prevalent in males (56%) than in females [34,41,42,43,44]. As expected, the cohort in this study consists of more males (59%) than females. This is in contrast to the general population in the region, which is evenly divided (50% male) [45]. It could also be that males are more exposed to noise, which makes them more prone to hearing loss, which in turn is associated with higher burden scores. In this study, 42% of the males reported noise exposure as a potential cause of their tinnitus as opposed to 15% of women. In turn, 23% of the male participants reported stress as a potential cause as compared to 48% of women. However, another consideration could be the willingness to take part in studies between men and women. 

Importantly, it should be noted that in this specific cohort hearing loss was highly prevalent. Hearing loss was associated with a higher tinnitus burden. This was as expected since hearing loss plays a prominent role in the pathophysiology of tinnitus [41]. There are several potential underlying causes of hearing loss which can be related to the development of tinnitus [46]. Recently, it has been shown that hearing aids can successfully treat tinnitus in participants with a hearing impairment [47]. In future studies, satisfaction with hearing aids could also be assessed to specify this outcome.

When considering life events, we hypothesized that life events may have an impact as they can be thought of as a source of stress in daily life. Indeed, it was observed that tinnitus burden was related to the amount of positive and negative life events someone experienced. However, both had a lowering impact indicating the more life events were reported the lower the tinnitus burden was. Being occupied with life events seems to result in distraction, taking focus away from the tinnitus. 

Furthermore, participants with depressive and anxiety symptoms experience a higher tinnitus burden. This is in line with previous reports [48,49,50,51]. Another study demonstrated that tinnitus is associated with depression prior to tinnitus [52] which indicates that treating a comorbid psychological disorder could already alleviate burden without having to affect the tinnitus itself. 

Additionally, participants with sleep issues had a significantly higher burden of tinnitus. It has been demonstrated by others that 60% of tinnitus participants meet strict diagnostic criteria for insomnia (DSM-IV-TR) [53] but only a very small subgroup of these patients were being treated for their insomnia [54]. For a personalized treatment of tinnitus patients, it is important to take sleep issues into account. Recently, it has been shown that solely treating sleep difficulties has a positive impact on the tinnitus burden [55,56]. 

Another factor associated with tinnitus burden is hearing impairment with the presence of hearing impairment being associated with higher tinnitus burden as had been shown in previous research [57].

### 4.2. Predictors of Tinnitus Burden over Time

Longer tinnitus duration predicts a lower tinnitus burden after one year. Despite this being a small reduction, it could imply a process of habituation over time. 

The perceived loudness of the tinnitus sound indicated a relationship between perceived loudness and burden, showing more burden with more perceived loudness. This relationship between perceived loudness and psycho-emotional burden has been shown before [58]. 

Even though anxiety and depression were significantly related to tinnitus burden in general, depression was not important in predicting changes in tinnitus burden over 12 months. Interestingly, the potential impact of anxiety on TQ after 12 months was a reduction in burden. It seems that if anxiety is high in the first period of tinnitus, this is frequently followed by a period of less burden.

Surprisingly, in this cohort, the tinnitus burden was not reduced in participants that underwent a treatment that is proven to be effective (hearing aids, masking, CBT or medication for psychiatric comorbidity) or any other treatment. In the Netherlands, the treatment guidelines for tinnitus advice first of all recommend hearing rehabilitation in persons with impaired hearing, secondly psychological care (in the form of CBT) and lastly treatment of comorbid psychiatric symptoms using psychiatric medication [59]. The other reported treatments were physiotherapy, acupuncture and neuromodulation techniques. It seems that the persons in this study were not responsive to these treatments, which could mean that current available treatments are unsatisfactory or that there was a ceiling effect for treatment. Namely, patients were partly recruited in a tertiary clinic and these persons could already have gone through treatment in different institutions.

The importance of sleep for both general health as well as chronic conditions is becoming more widely recognized [60]. It had been hypothesized that tinnitus and insomnia share several underlying physiological mechanisms (i.e., hyperarousal of the sympathetic nervous system). Additionally, aspects of the burden imposed by both conditions overlap significantly, i.e., compromised daily living, depression and anxiety [61]. Therefore, the negative impact of sleep issues on tinnitus can be expected. Moreover, it can be frustrating when tinnitus is perceived as the source of the sleeping issues. It has also been shown that reducing sleep disturbances can have an impact on tinnitus burden [55]. 

The amount of life events had a positive (lowering) effect on tinnitus burden. It could be that being occupied by life events puts the tinnitus burden in a different perspective. In addition, as expected, the amount of negative life events had a negative effect on tinnitus burden after 12 months. They seem to cause more stress and therefore result in a higher burden score over time. 

The environmental sub score of the QoL measure also had a positive impact on the tinnitus burden over time. It could be hypothesized that the environmental sub score of the WHO-QoL Bref is related to the social economic status of the participant and that persons with a higher social economic status have more access to certain protective measures as well as that they may have a higher education level.

Hearing impairment in the final model was associated with higher tinnitus burden after 12 months. This could be to do with the fact that we do have objective information about hearing impairment and its rehabilitation which we discuss in the limitations. 

### 4.3. Limitations

Our work has several notable limitations, largely arising from the sample population. The sample is a selected tinnitus population. In addition, the sample was heterogeneous across several factors. It is thus possible that non-significant relationships or effects we report could be significant in larger or less heterogeneous samples. Moreover, it could be argued that reporting on life events retrospectively may not be the optimal format. One suggestion for the future is to use a more focused approach such as ecological momentary assessment to combine active daily life stress with tinnitus burden [62] (Lourenco et al., 2022).

Secondly, no objective measurements of the hearing quality of the participants were available, as we did not have access to the participants’ medical histories. This is a limitation as there is a large group of tinnitus patients with comorbid hearing loss. Additionally, in future research, it can be of interest to also monitor the participants’ satisfaction with their hearing aids. 

Lastly, the self-report character of the study means we were not provided with objective reports on the participants’ medical background and hearing status. In order to achieve this, accessing the participants’ patient files would be necessary but this was beyond the scope of this study. Therefore, the reported comorbidities may not be entirely accurate; however, we do assume patients are sufficiently knowledgeable about their own health to just report on it. 

## 5. Conclusions

Within a help-seeking tinnitus population, several factors were found that positively or negatively contributed to tinnitus burden over time. 

Current treatment options seem not to be sufficient for an overall tinnitus burden decrease. The factors found in this study can be of help to improve treatment and form a more patient-specific treatment strategy.

## Figures and Tables

**Table 1:** Demographics of the population at baseline, numbers and percentages are given.

Variable	*n* (%)	
**Participants (*n*)**	383	
Men (*n*)	231	(60.3%)
Age (in years)	57	(range: 18–88)
**Highest education**		
Low	197	(51.4%)
High	186	(48.6%)
**Job status**		
Employed		
*part-time*	*50*	*(13.1%)*
*full-time*	*134*	*(35.0%)*
Unemployed		
*not searching for a job*	*13*	*(3.4%)*
*searching for a job*	*12*	*(3.1%)*
Not working due to chronic illness	22	(5.7%)
*Not due to tinnitus*	*15*	*(3.9%)*
*Due to tinnitus*	*9*	*(1.8%)*
Disqualified for work	10	(2.6%)
Retired	101	(26.4%)
**Marital status**		
Married with children	156	(40.7%)
Married	99	(25.8%)
Living with partner	44	(11.5%)
Single	44	(11.5%)
In a relationship but living apart	16	(4.2%)
Living with partner and children	18	(4.7%)
Single parent	6	(1.6%)
**Family size**		
1 person	54	(14.1%)
2 persons	203	(53.0%)
3–4 persons	106	(27.7%)
≥5 persons	20	(5.2%)

**Table 2:** Description of the tinnitus complaints at baseline, numbers and percentages are given unless mentioned differently.

**Variable**	***M* (*SD*)**	
Tinnitus Questionnaire score at T0 (baseline) Tinnitus Questionnaire score at T1 (6 months) Tinnitus Questionnaire score at T2 (12 months)	34 (18) 32 (18) 32 (18)	
**Variable**	***n* (%)**	
**Duration** (T0)		
Acute (<6 months)	39	(10.2%)
Chronic (>6 months)	344	(89.8%)
**Start event**		
Combination of causes	126	(32.9%)
Single causes		
No clear cause	88	(23.0%)
Noise exposure	53	(13.8%)
Stress	41	(10.7%)
Change in hearing	18	(4.7%)
Other medical cause	18	(4.7%)
Infection	15	(3.9%)
Feeling of pressure or stuffed ear	10	(2.6%)
Neck trauma	5	(1.3%)
Head trauma	5	(1.3%)
Change in air pressure	4	(1.0%)
**Onset**		
Sudden	187	(48.8%)
Gradual	144	(37.6%)
Not sure	52	(13.6%)
**Burden presence**		
Daily or almost daily	360	(94.0%)
Weekly or more	23	(6.0%)
**Consistency**		
Constant	362	(94.5%)
With breaks	21	(5.5%)
**Pitch**		
High	259	(67.6%)
Medium	93	(24.3%)
Low	19	(5.0%)
NA	12	(3.1%)
**Burden severity**		
Severely	158	(41.3%)
Moderately	120	(31.3%)
Slightly	91	(23.8%)
Not at all	12	(3.1%)
Do not know	2	(0.5%)
**Type of tinnitus sound**		
One sound	219	(57.2%)
More than one sound	164	(42.8%)
**Description of tinnitus sound**		
Tone	227	(59.3%)
Noise	109	(28.5%)
Crickets	25	(6.5%)
Rumbling	15	(3.9%)
Other	6	(1.6%)
Music	1	(0.3%)
**Fluctuations in tinnitus sound**		
Varies sometimes	209	(54.6%)
Stable	109	(28.5%)
Varies always	65	(17.0%)
**Rhythm of tinnitus**		
Not rhythmic	275	(71.8%)
Other	35	(9.1%)
I don’t know	30	(7.8%)
Following the rhythm of the heart	28	(7.3%)
Following the movement of the head, neck, jaw or facial muscles	14	(3.7%)
Following the breathing	1	(0.3%)
**Location of tinnitus**		
Bilateral	214	(55.9%)
*Equal*	*76*	*(19.8%)*
*Lateralized to the left*	*73*	*(19.1%)*
*Lateralized to the right*	*65*	*(17.0%)*
Unilateral	95	(24.8%)
*Left ear*	*55*	*(14.4%)*
*Right ear*	*40*	*(10.4%)*
Inside the head	56	(14.6%)
Other namely…	15	(3.4%)
I don’t know	1	(0.3%)
**Perceived hearing**		
Normal hearing	146	(38.1%)
Impaired hearing	237	(61.9%)
*Impairment measured by healthcare professional*	*211*	*(55.1%)*
*Wearing hearing aids*	*119*	*(31.1%)*
**Location of hearing impairment**		
Bilateral *Equal*	179 (46.7%) *63 (16.4%)*	
*Lateralized left*	*61 (15.9%)*	
*Lateralized right*	*55 (14.4%)*	
Unilateral *Left*	58 (15.1%) *27 (7.0%)*	
*Right*	*31 (8.1%)*	
**Hearing aids**		
Conventional hearing aids	108	(28.2%)
Sound generator	13	(3.4%)
Cochlear implant	7	(1.8%)
Bone conduction device	4	(1.0%)
**Treatment during studied period**		
Only proven-effective treatment(s)	39	10.2%
Psychological treatment *	*28*	
Audiological treatment *	*14*	
Psychiatric medication *	*8*	
Combining proven and other treatments	21	(5.5%)
Other treatment(s) only	50	(13.1%)
No treatment	273	(71.3%)

* participants could report multiple treatment types, therefor only absolute numbers reported.

**Table 3:** Regression model for TQ total scores.

Outcome Variable: TQ Total				
Effect	*B*	95% CI		*p*
		LL	UL	
Time (per 6 months)	−0.910	−1.669	−0.150	0.019
Sex	−2.260	−3.543	−0.978	<0.001
Age in years	0.010	−0.042	0.062	0.708
Education (high)	−3.510	−4.815	−2.205	<0.001
Amount of positive life events	−0.290	−0.550	−0.029	0.029
Amount of negative life events	−0.358	−0.573	−0.144	0.001
HADS anxiety (0–21)	0.559	0.347	0.771	<0.001
HADS depression (0–21)	1.009	0.782	1.236	<0.001
Sleep issues (present)	3.758	2.375	5.142	<0.001
VAS tinnitus loudness (0–100)	0.384	0.351	0.416	<0.001
Hearing impairment at baseline (present)	2.109	0.747	3.471	0.002
Proven effective treatment	4.654	2.834	6.474	<0.001
Other treatments	2.180	0.521	3.840	0.010

TQ = Tinnitus Questionnaire ranges from 0 to 84; HADS = Hospital Anxiety and Depression Scale ranges from 0 to 21. Treatments proven effective include audiological care, cognitive behavioral therapy and psychiatric medication.

**Table 4:** Regression models. (A) Univariable regressions with TQ after 12 months as outcome variable. (B) The final model of the multivariable regression after backward elimination with TQ after 12 months as the outcome variable.

(A) Univariable regression				
Effect	*B*	95% CI		*p*
		LL	UL	
TQ at baseline	0.687	0.643	0.731	<0.001
Age at baseline	0.124	0.044	0.204	0.002
Sex	−2.145	−4.262	−0.028	0.047
Education level (high/low)	−8.279	−10.298	−6.260	<0.001
Tinnitus duration (in years)	0.010	−0.089	0.109	0.847
VAS loudness at baseline	0.408	0.365	0.452	<0.001
HADS anxiety at baseline	1.388	1.156	1.620	<0.001
HADS depression at baseline	1.871	1.649	2.093	<0.001
Followed treatment (T0–T2)	13.127	10.964	15.290	<0.001
Proven effective treatment (T0–T2)	15.703	12.998	18.408	<0.001
Other treatment (T0–T2)	10.421	7.820	13.021	<0.001
Sleep issues present (T0–T2)	11.980	10.024	13.935	<0.001
Total amount of life events (T0–T2)	−0.067	−0.306	−0.173	0.585
Positive life events	−1.021	−1.424	−0.619	<0.001
Negative life events	0.783	0.449	1.116	<0.001
QoL general at baseline	−1.943	−2.213	−1.672	<0.001
QoL physical health at baseline	−2.590	−2.891	−2.289	<0.001
QoL psychological health at baseline	−2.971	−3.389	−2.553	<0.001
QoL social relations at baseline	−1.643	−2.023	−1.264	<0.001
QoL environment at baseline	−2.505	−2.955	−2.055	<0.001
Hearing impairment at baseline	6.395	4.292	8.499	<0.001
(B) Multivariable regression (final model)				
Effect	*B*	95% CI		*p*
		LL	UL	
TQ at baseline	0.553	0.487	0.619	<0.001
Tinnitus duration (in years)	−0.129	−0.203	−0.054	<0.001
VAS loudness at baseline	0.055	0.007	0.103	0.025
HADS anxiety at baseline	−0.401	−0.625	−0.177	<0.001
Proven-effective treatment (T0–T2)	6.154	4.014	8.293	<0.001
Other treatment (T0–T2)	4.069	2.092	6.046	<0.001
Sleep issues present (T0–T2)	3.977	2.399	5.555	<0.001
Total amount of life events	−0.413	−0.671	−0.156	0.002
Negative life events	0.713	0.343	1.084	<0.001
QoL environment at baseline	−0.665	−1.059	−0.271	<0.001
Hearing impairment at baseline	1.987	0.383	3.591	0.015

TQ = Tinnitus Questionnaire ranges from 0 to 84; HADS = Hospital Anxiety and Depression Scale ranges from 0 to 21; QoL = quality of life.

## Data Availability

The data presented in this study are available on request from the corresponding author due to privacy reasons.

## Appendix A

**Overview of the questionnaires used:**
  - Demographics (11 questions): basic demographic information (i.e., age, sex, education, job status, marital status and family size).
  - General health (10 questions): these questions provide a background about comorbid disorders and burden of other health related issues. Questions were taken from the ESIT-SQ a self-reported tinnitus-relevant history questionnaire for standardized collection of information from both tinnitus and non-tinnitus populations [63].
  - Tinnitus characteristics (12 questions): a description of the type of tinnitus the participant experience (questions taken from the ESIT-SQ [63]).
  - Perceived hearing (5 questions): provides insight in how the participants perceived their hearing in order to relate possible changes in tinnitus and hearing.
  - Help seeking behavior (3 questions): gives insight in the amount and type of help the participant sought in the past year (questions taken from the ESIT-SQ [63]).
  - Tinnitus Questionnaire (TQ) (52 questions): this validated questionnaire consists of 52-items answered on a 3-point scale to measure tinnitus severity. This renders a severity score (0-84) which can divide the population in four categories based on severity, a higher scores means more tinnitus suffering. In standard care patients are offered an audiological or psychological intervention if the TQ-score is 47 or higher [15,16].
  - Visual Analog Scale (VAS) [18,19] Burden (on a slider 0–100) (1 question): the participant is asked to indicate the burden they experience from their tinnitus on a scale from 0 (not burdensome at all) to 100 (extremely burdensome). When moving the slider the selected number pops up above the slider.
  - Visual Analog Scale (VAS) [18,19] Loudness (slider 0–100) (1 question): the participant is asked to indicate the loudness of their tinnitus on a scale from 0 (not loud at all) to 100 (extremely loud). When moving the slider the selected number pops up above the slider).
  - Life events (50 questions): allows for separate assessment of positive and negative life events. It also asks for individual ratings of the impact of events (based on the Life Experiences Survey [64].
  - Hospital Anxiety and Depression Scale (HADS) (14 questions): this 14-item questionnaire evaluates the psychological suffering on two scales (anxiety and depression). Each scale has a maximum score of 21 points and the results can be divided in four categories based on severity [20,21]. A higher scores means more anxious or depressive symptoms.
  - WHO-QOL BREF (26 questions): this 26-item questionnaire evaluates the quality of life in four domains (physical health, psychological, social relationships and environment) on a 6-point likert-scale [22]. A higher score represents a higher quality of life.

## Appendix B

**Prevalence of comorbidities reported by the participants.**

**Variable**	***n***
Procedures	201
Lumbar puncture	89
Dental surgery	73
Other surgical procedures	67
Ear surgery	37
Chemotherapy	9
Brain or spinal surgery	6
Radiation of head and-or neck	6
Electroconvulsive therapy	0
Sleep	191
Waking up at night	140
Trouble falling asleep	111
Miscellaneous	136
Misbalance	72
Reflux	64
Globus feeling	20
Anemia	9
Cardiovascular	132
High blood pressure	79
Other	34
Heart attack	17
Low blood pressure	18
Psychologic	105
Depression	60
Anxiety	43
Other	37
Dental and mandibular	99
Dental issues	82
Temporomandibular pain	31
Metabolic	94
Elevated cholesterol	62
Thyroid issues	25
Diabetes	13
Other	4
Rheumatologic/immunologic	61
Other	30
Fibromyalgia	20
Rheumatoid arthritis	14
Neurologic	54
Other	46
Stroke	5
Meningitis	3
Epilepsy	2
Multiple sclerosis	1
Dementia	0
Infectious	21
Other	17
Lyme disease	3
Syphilis	1
HIV	0

## Appendix C

**Sankey diagram showing the progression of tinnitus burden over time (A) (expressed by tinnitus questionnaire grades: (B) TQ 0–30 little burden, (C) TQ 31–46 mild burden, (D) TQ 47–59 severe burden, (E) TQ 60–84 very severe burden).**
